# Virtual consultations: delivering outpatient clinics in paediatric surgery during the COVID-19 pandemic

**DOI:** 10.1186/s43159-020-00060-w

**Published:** 2020-11-30

**Authors:** A. M. Charnell, E. Hannon, D. Burke, M. R. Iredale, J. R. Sutcliffe

**Affiliations:** 1 Department of Paediatric Surgery, Leeds Children’s Hospital NHS Trust, Leeds, UK; 2 Leeds Institute of Medical Education, University of Leeds, Leeds, LS2 9JT UK

**Keywords:** Remote clinics, Telemedicine, Surgical outpatient clinics, Video consultations, Surgical education

## Abstract

**Background:**

The COVID-19 pandemic has resulted in many changes to clinical practice, including the introduction of remote clinics. Those familiar with remote clinics have reported benefits to their use, such as patient satisfaction and cost benefits; however, ongoing challenges exist, including delivering optimal patient-centred care. As a tertiary paediatric surgery unit in the UK, completing remote clinics was a new experience for most of our surgical team. We completed a service evaluation early into the COVID-19 pandemic aiming to define and address issues when delivering remote clinics in paediatric surgery. Remote clinics were observed (telephone and video), with follow-up calls to families following the consultations.

**Results:**

Eight paediatric surgeons were observed during their remote clinics (telephone *n* = 6, video *n* = 2). Surgeons new to remote clinics felt their consultations took longer and were reluctant to discharge patients. The calls did not always occur at the appointed time, causing some upset by parents. Prescription provision and outpatient investigations led to some uncertainty within the surgical team. Families (*n* = 11) were called following their child’s appointment to determine how our remote clinics could be optimised. The parents all liked remote clinics, either as an intermediate until a face-to-face consultation or for continued care if appropriate.

Our findings, combined by discussions with relevant managers and departments, led to the introduction of recommendations for the surgical team. An information sheet was introduced for the families attending remote clinics, which encouraged them to take notes before and during their consultations.

**Conclusions:**

There must be strong support from management and appropriate departments for successful integration of remote clinics. Surgical trainees and their training should be considered when implementing remote clinics. Our learning from the pandemic may support those considering integrating remote clinics in the future.

## Background

The pandemic has enforced rapid changes to clinical practice, including outpatient care. In the UK, all consultations must be delivered by telemedicine during the pandemic unless clinical or practical reasons dictate otherwise [1]. Many clinical institutions worldwide have adopted a similar practice.

### Remote clinics pre-COVID

Remote consultations (video or telephone) are not a new phenomenon and can be preferable and more convenient for patients with cost benefits for the institution [2, 3]. Experience within surgical specialities pre-COVID is limited; orthopaedics had utilised virtual fracture clinics for many years leading to reduced costs, lower staffing levels, and reduced need for follow-ups with appropriate patient selection [4].

Within paediatric surgery, Smith and colleagues have used remote review of distant patients with acute surgical pathologies, particularly burn patients, with local clinicians in attendance for remote inpatient reviews [5]. They also utilise remote consultations to review children referred for elective surgeries, such as undescended testes. The children attend for face-to-face review if appropriate with a same-day operation if required.

### Remote clinics in the COVID era

Descriptions of remote surgical clinic use during the pandemic are emerging, and guidance has arisen regarding remote clinics; however, reflections focus on the clinician completing the clinics, without considering other key-players [6]. Grenda and colleagues describe their transition to video remote outpatient clinics in adult thoracic surgery [7]. They have moved entirely to remote clinics for new referrals to determine management plans. Post-operatively, they use remote consultations to assess their patients and consider the need for further face-to-face consultations [7].

Although triage and clinical prioritisation in remote clinics have been considered, focus to date is on institutional guidance rather than the provision of surgeon- or patient-centred advice.

### Supporting the transition to remote clinics

Since many teams have integrated remote clinics with pace, uncertainties exist regarding the potential to affect outcomes for patients and undermine the confidence of staff [6]. In this tertiary paediatric surgery department, consultant surgeons and their trainees traditionally reviewed regional patients face-to-face. Although some consultants utilised remote consultations through their clinics, experience was limited. We aimed to define and address issues that could enhance the ability to deliver remote clinics during the pandemic.

## Methods

A service evaluation was undertaken to consider what practical support could be given to clinical staff and parents to enhance consultations. One investigator observed remote clinics with paediatric surgeons, and follow-up discussions were held with surgeons, clinic staff, managers, key departments, and patients. A range of paediatric surgery clinics (colorectal, upper gastrointestinal, hepatobiliary, and urology) were selected from those completed in the first 2 weeks of the transition, including as many surgeons and presentations as possible. During this time, only follow-up patients were reviewed. Those excluded were clinics completed from the clinicians’ homes. Ethical approval was sought from the Medical Research Council but was not required for this service evaluation. Local quality improvement permissions were obtained.

## Results

### Observation of clinics

At the time of evaluation, remote clinics were newly introduced and almost entirely consultant-led. Eight different consultant surgeons were observed. Remote clinics were completed within the outpatient clinic rooms with the standard physical set-up, although some were completed from home. A small number of face-to-face clinics did take place. These normally occurred when it was felt that the patient required review, such as following investigation or when post-operative concerns arose.

Within remote clinics, paper notes were initially available, but most clinicians used the patient’s electronic health record instead. Later, the Trust made the paper notes only available when requested. Most surgeons delivered telephone consultations (*n* = 6), although some utilised video (*n* = 2). When video consultations were completed, they were performed using Attend Anywhere video-conferencing software, with details sent to the patient beforehand.

Consultants for whom phone consultations were new often reported that consultations were not as useful as face-to-face clinics and that clinics took longer. There was a reluctance to discharge patients by those surgeons new to remote clinics. These hesitancies appeared reduced in those more familiar with remote clinics, suggesting the cause is unfamiliarity.

As with face-to-face clinics, maintaining the planned timing of consultations was sometimes difficult. One patient was called 30 min early and missed the call. Although the surgeon called the patient back later in the clinic, the parent had already called the secretary to express frustration, asking for the mobile number of the clinician. Although most patients understand that the timing cannot be exact, it may be necessary to emphasise the timings may vary due to current circumstances.

A recurrent observation concerned prescriptions; the initial lack of guidance meant that each surgeon prescribed medications differently during remote clinics. Some wrote letters to the general practitioners, and others left prescriptions in the department for the patient to collect. Early intra-departmental discussions allowed families to collect their prescriptions and medicines directly from the on-site pharmacy if needed.

### Discussions with the parents

Remote consultations are also new to many parents and children. Phone calls were made to 11 sets of parents following their remote appointments (three video consultations and eight telephone). Parents who had phone appointments stated a preference for phone and did not feel the video would have enhanced their appointment. Those who had video consultations preferred video, especially when they had not met the consultant before; however, one mum said her son was horrified at seeing the surgeon on camera and would not participate in the conversation. Although these remote consultations began due to COVID-19, parents were very positive about their experience, and some made clear that they preferred them (“it’s the way forward”). Reasons included not having to park and not having to look after restless children in waiting rooms. Others viewed them as an intermediate solution until their next face-to-face appointment.

Parents were concerned that they did not know what information their surgeon wanted before the virtual clinic, emphasising it would be useful to know in advance if the consultant might want any information, such as the child’s weight. One mother had run upstairs to weigh her child on request, but was apologetic for keeping the consultant waiting, stating: “I was conscious of taking up their time”.

Parents were asked about their ability to ask questions and retain information from their consultation. Some said they had only thought of questions after, as they were conscious that the surgeons were busy and thought they should rush through. When asked, all parents agreed that being advised about having a pen and paper handy in preparation for the session would be useful.

### Interventions

**Fig. 1:**
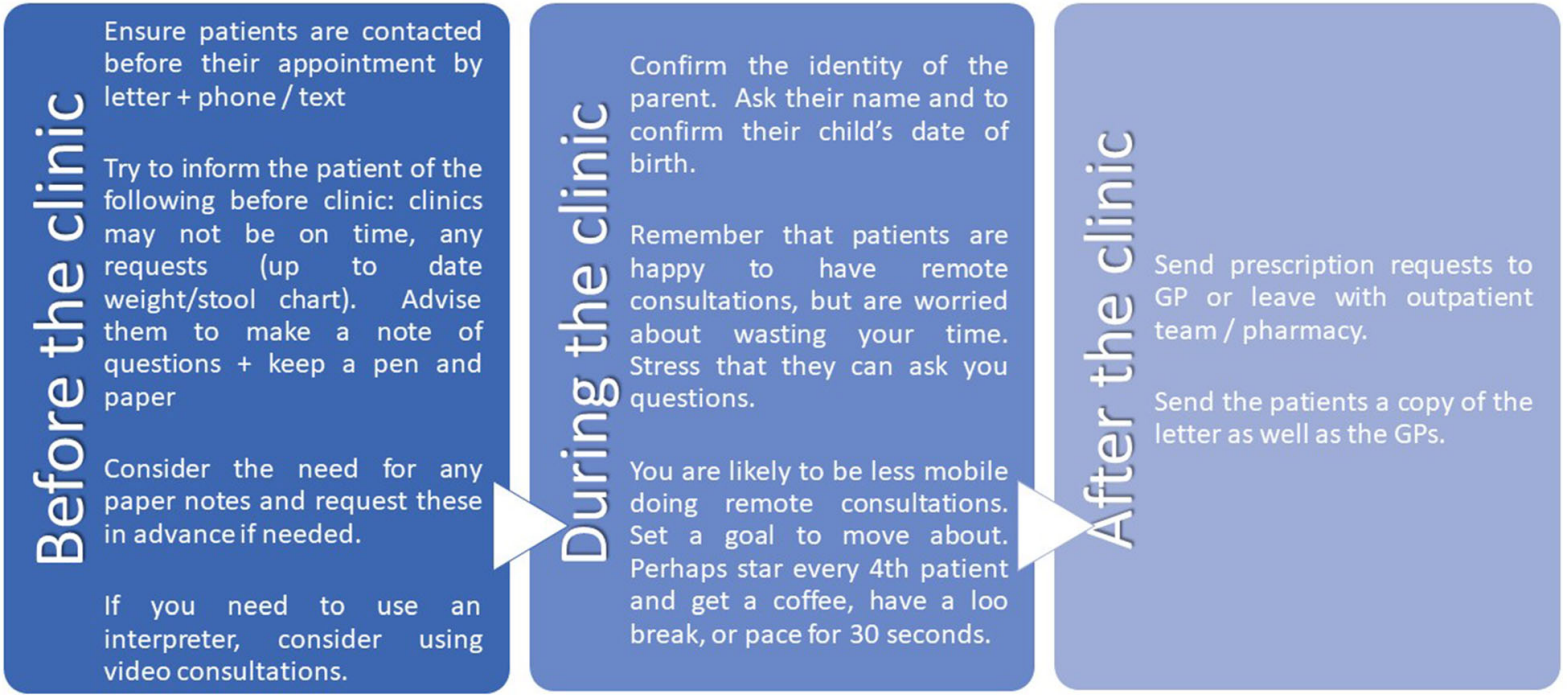
Key recommendations for surgical staff

**Fig. 2:**
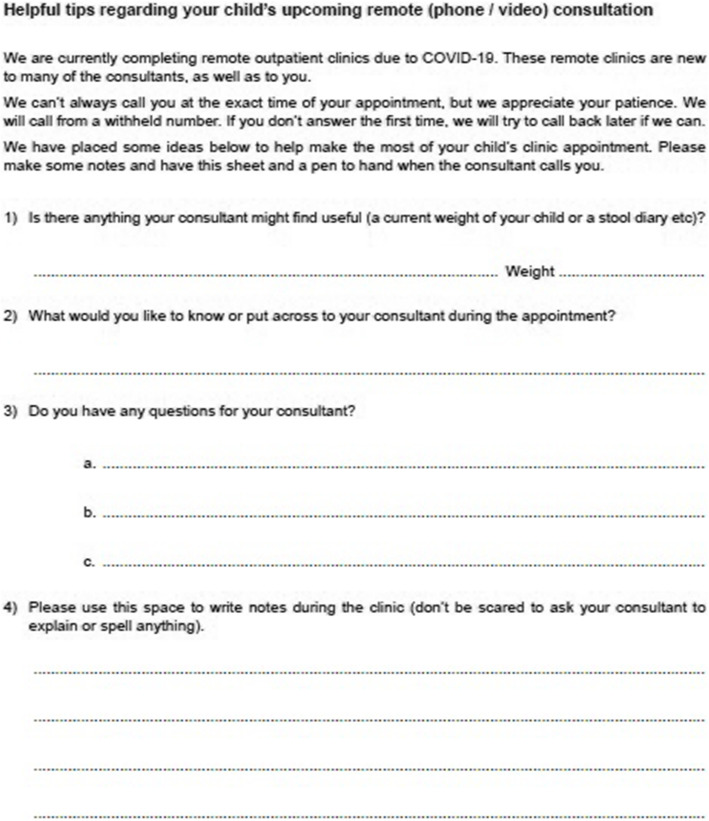
Letter sent to parents in advance of remote clinic appointments

Based on our findings, we have implemented two primary support interventions: a list of key findings for surgical staff (Fig. 1) and a letter for parents (Fig. 2).

## Discussion

We report our early experience of remote clinics to deliver outpatient care during COVID-19. While this evaluation was specific to our department and development is ongoing, we believe some generalisable messages may be useful to other groups. Particular areas of focus included information to send patients attending clinics, confirming the identity of the parents, allowing time for discussion, moving around the room between patients, and having strategies for interventions following the clinic.

Attending a face-to-face clinic in a tertiary centre is both costly and time-consuming for parents taking time from work and children from school. Conversely, access to technology, especially for video consultations, may be difficult for families without appropriate devices and Internet access. Overall, remote clinics lead to reduced failed attendances (7% vs 27%) and can support triaging those that need face-to-face review and operations [5]. Personal communication with Smith and co-authors emphasises the need for good communication with families at all stages regarding face-to-face reviews. Patients are happy to travel across Australia after their remote clinic for face-to-face consultation, which may result in a same-day operation, or not, as appropriate.

Regarding costs, under the Aligned Incentive Contract, our Trust receives the same financial amount for remote as face-to-face consultations. Early on, it is difficult to determine whether remote clinics are cost effective, but other benefits of remote consultations are clear and were emphasised by our patients: reduced time off work and not needing to park and spend time in waiting rooms.

At the time of evaluation, virtual clinics were almost entirely for follow-up patients and consultant-delivered. We have now introduced trainee-delivered clinics and we also review selected new patients remotely. Remote consultations have resulted in a massive transition for many surgeons: from expert to learner. Consideration of how trainees might deliver care in these clinics and use them educationally is warranted. A ‘washup’ at the end of the clinic should be timetabled to discuss clinical or learning points. It is important to ensure the consultant surgeons, and their patients, feel confident enough in remote clinics to support trainees within the clinic’s community of practice. Since surgical trainees typically complete an average of three clinics per week [8], there is also an evident service provision element to be considered if virtual clinics become the norm. Through multi-layered support, we can provide optimal patient-centred care.

Clinician concerns on the care provided during the shift to remote clinics are likely to decrease with time and guidance, which we continue to evaluate. Current areas of focus are determining those patients not suitable for remote consultations, pathway and patient-specific considerations (i.e. a definite need to be examined), confidence in discharging patients, and our inability to assess factors such as family dynamics. The European Association of Urology guidelines panel for paediatric urology suggest that photographic documentation may be uploaded to the child’s file in advance if needed [9]. Photographs may help to determine healing of post-operative wounds and consider skin changes; these may support the decision of whether a patient should be reviewed face-to-face.

We have set up a secure email so that families can send images, but if examination is required, patients are seen face-to-face. Due to the intimate nature of consultations, the urology team review all new patients face-to-face. It is difficult to determine information such as family dynamics over the phone, so close conversation with the multidisciplinary team, including the general practitioner, is paramount, especially if any concerns are suspected.

Moving forward, we can utilise our learning and consider the necessity of face-to-face consultations, while appreciating the benefits of remote clinics for patients, parents, and clinical staff.

## Conclusions

Those considering integrating remote clinics into their surgical practice should have strong support from management and appropriate departments, such as pharmacy and radiology, for successful integration of remote clinics. Initially, our remote clinics were consultant-led, but surgical trainees now also complete remote consultations, and their learning should be considered when implementing remote clinics. The reflections on our experiences from the pandemic may support those considering integrating remote clinics in the future.

## Data Availability

Data sharing is not applicable to this article as no datasets were generated or analysed during the current study.
